# Oral yeast colonization throughout pregnancy

**DOI:** 10.4317/medoral.21413

**Published:** 2017-02-04

**Authors:** Rute Rio, Liliana Simões-Silva, Sofia Garro, Mário-Jorge Silva, Álvaro Azevedo, Benedita Sampaio-Maia

**Affiliations:** 1 Faculdade de Medicina Dentária, Universidade do Porto; 2 Universidade Católica Portuguesa - Campus Viseu; 3 I3S-Instituto de Investigação e Inovação em Saúde, Universidade do Porto; 4 INEB-Instituto Nacional de Engenharia Biomédica, Universidade do Porto; 5 Faculdade de Medicina, Universidade do Porto; 6 Escuela de Medicina, Universidade da Costa Rica, Costa Rica; 7 EPIUnit-Instituto de Saúde Pública, Universidade do Porto

## Abstract

**Background:**

Recent studies suggest that placenta may harbour a unique microbiome that may have origin in maternal oral microbiome. Although the major physiological and hormonal adjustments observed in pregnant women lead to biochemical and microbiological modifications of the oral environment, very few studies evaluated the changes suffered by the oral microbiota throughout pregnancy. So, the aim of our study was to evaluate oral yeast colonization throughout pregnancy and to compare it with non-pregnant women.

**Material and Methods:**

The oral yeast colonization was assessed in saliva of 30 pregnant and non-pregnant women longitudinally over a 6-months period. Demographic information was collected, a non-invasive intra-oral examination was performed and saliva flow and pH were determined.

**Results:**

Pregnant and non-pregnant groups were similar regarding age and level of education. Saliva flow rate did not differ, but saliva pH was lower in pregnant than in non-pregnant women. Oral yeast prevalence was higher in pregnant than in non-pregnant women, either in the first or in the third trimester, but did not attain statistical significance. In individuals colonized with yeast, the total yeast quantification (Log10CFU/mL) increase from the 1st to the 3rd trimester in pregnant women, but not in non-pregnant women.

**Conclusions:**

Pregnancy may favour oral yeast growth that may be associated with an acidic oral environment.

**Key words:**Oral yeast, fungi, pregnancy, saliva pH.

## Introduction

Oral microorganisms are capable of passing through oral mucous membranes, spreading to different body sites and causing systemic or focal infections (1). Also, recent studies described that placenta may harbour a unique microbiome (2,3), and apparently this microbiome is more similar to the oral cavity than to the vagina microbiome (2). In fact, it is known that microorganisms may reach the uterus through a haematogenous route (4,5) and that maternal microbiomes from different body sites, including oral, vaginal, gut, cervical, and even the placenta itself, may influence pregnancy outcomes (6). Also, several studies suggested that the presence of periodontal pathogenic microorganisms or their by-products in the intrauterine environment stimulate an foetal immune and inflammatory response that may be responsible for the increased risk of preterm birth and low birth weight (7-10).

During pregnancy, yeast colonization of vagina is known to increase (11) and appears to be associated with an increased risk of pregnancy complications and preterm birth (12-15). Although the major physiological and hormonal adjustments observed in pregnant women lead to biochemical and microbiological modifications of the oral environment (16), very few studies evaluated the changes that the oral microbiota may suffer throughout pregnancy. So, the aim of our study is to evaluate oral yeast colonization throughout pregnancy and compare this colonization rate with the colonization in non-pregnant women.

## Material and Methods

A sample composed by 30 pregnant women attending for routine examination to the outpatient clinic of the Obstetrics and Gynaecology department of Arrábida Hospital, Porto, Portugal, was evaluated in the 1st and in the 3rd trimester of pregnancy in a longitudinal study. The control group comprise 30 non-pregnant women aged between 18 to 40 years old attending the same hospital on routine clinical examination. Due to hospital change, three pregnant and five controls dropped out from the study. Exclusion criteria were high-risk pregnancy, having less than 16 teeth, being menopausal, drug addiction, and presenting compromising systemic or oral disease.

The research protocol was in compliance with Helsinki Declaration and was approved by the ethics committees of the Arrábida Hospital, Porto, Portugal. An informed, free and clear consent was provided for, and was signed by all participants. Confidentiality of all information was guaranteed on data storage and processing stages.

During the first visit a questionnaire was applied inquiring about age, academic degree, smoking and oral hygiene habits.

Saliva was collected and stored as previously described (17,18). In brief, unstimulated whole saliva was collected by spit into a sterile plastic container over a 5-min period, the salivary pH was measured using pH indicator paper (5.0-8.0, Duotest, Germany), and saliva for microbiological analysis was stored frozen at -80°C in a 1:1 proportion with Brain Heart Infusion broth (Cultimed, Barcelona, Spain) with 15% glycerol.

The saliva samples were rapidly defrosted in a 37°C water bath, mixed well and serially diluted up to 10-2 with 0.9% sterile NaCl solution. The samples were immediately plated in triplicate on Sabouraud agar (Cultimed, Barcelona, Spain) supplemented with chloramphenicol to evaluate the presence of fungi. The Sabouraud agar was incubated aerobically for 48 h at 37°C. Colonies were counted, and the results were expressed in Log10 of colony forming units per mL of saliva (Log10 CFU/mL) (19,20). Patients who presented <50 CFU/ml were considered low yeast carriers; patients who presented between 50 and 400 CFU/ml were considered moderate yeast carriers; and patients who presented >400 CFU/ml were considered high yeast carriers (21).

The statistical analyses were performed using the statistical analysis software Statistical Package for Social Sciences (SPSS) 21.0 for MAC OS. When appropriate, the chi-square independence test was used to analyse hypotheses regarding the categorical variables; the Mann-Whitney U Test or Wilcoxon Signed Ranks Test were used for the continuous variables. It was considered a significance level of 0.05.

## Results

As illustrated in  Table 1, both groups of pregnant and non-pregnant women were similar regarding the socio-demographic characteristics, such age (p=0.748) and level of education (*p*=0.386), as well as smoking (*p*=0.270) and oral hygiene habits (*p*=0.145). Neither pregnant nor non-pregnant woman presented clinical signs of candidiasis.

**Table 1:**
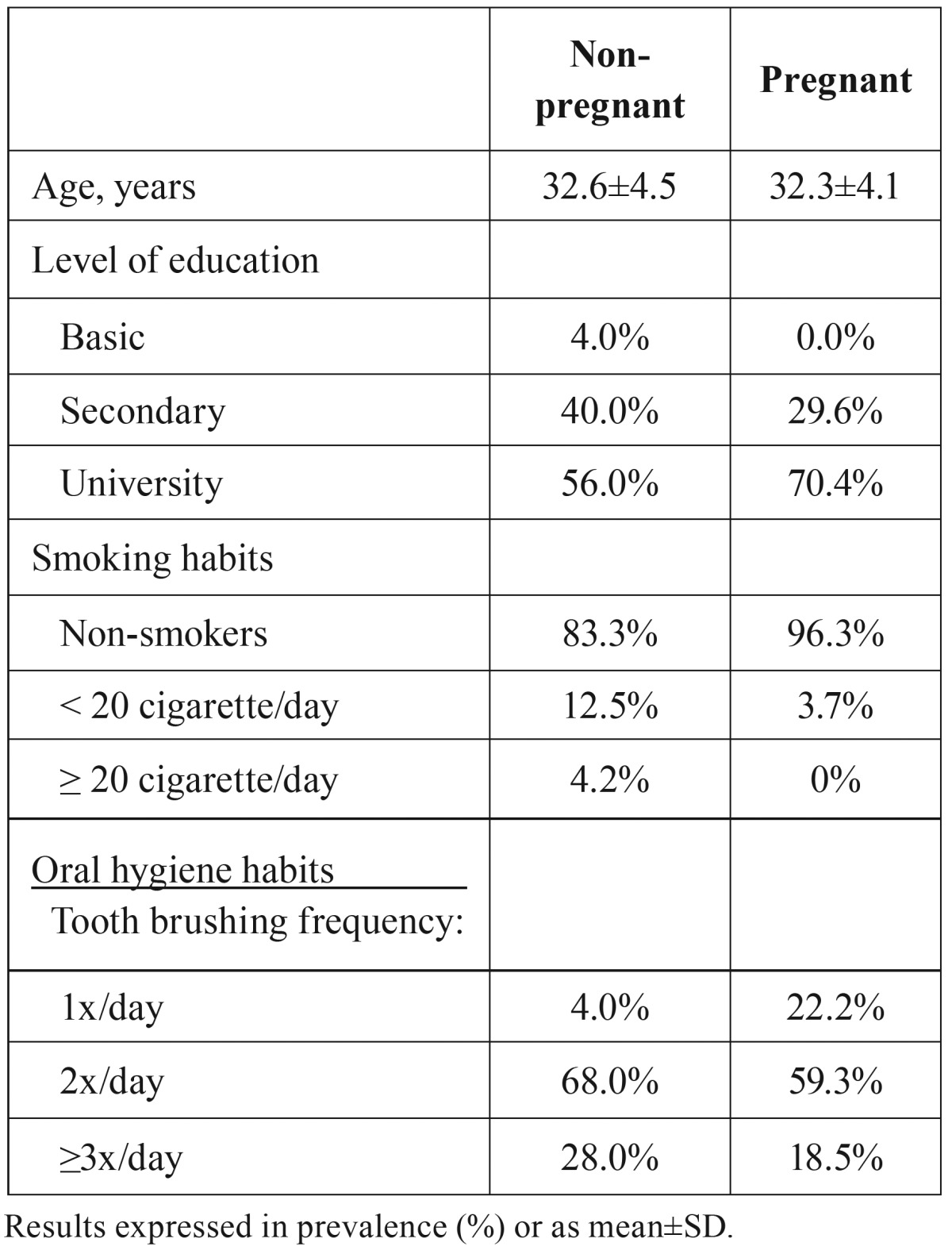
Age, level of education, smoking habits and oral hygiene habits of pregnant and non-pregnant women.

Saliva flow rate did not change from the first to the third trimester in both pregnant (1.86±0.49 vs. 1.89±0.40, *p*=0.776) and non-pregnant (1.71±0.46 vs. 1.78±0.48, *p*=0.318) women. Also, no differences were observed between pregnant and non-pregnant women regarding saliva flow rate at both time points (1stT: *p*=0.304; 3rdT: *p*=0.457). Saliva pH did not change from the first to the third trimester in both pregnant (6.69±0.35 vs. 6.73±0.28, *p*=0.433) and non-pregnant (7.04±0.28 vs. 7.03±0.24, *p*=0.782) women, but saliva pH was lower in pregnant than in non-pregnant women either in the first (*p*<0.001) or in the third (*p*<0.001) trimester.

Oral yeast prevalence was higher in pregnant than in non-pregnant women, either in the first or in the third trimester ( Table 2), although no statistical significance was found (1stT: *p*=0.114; 3rdT: *p*=0.071).

**Table 2:**
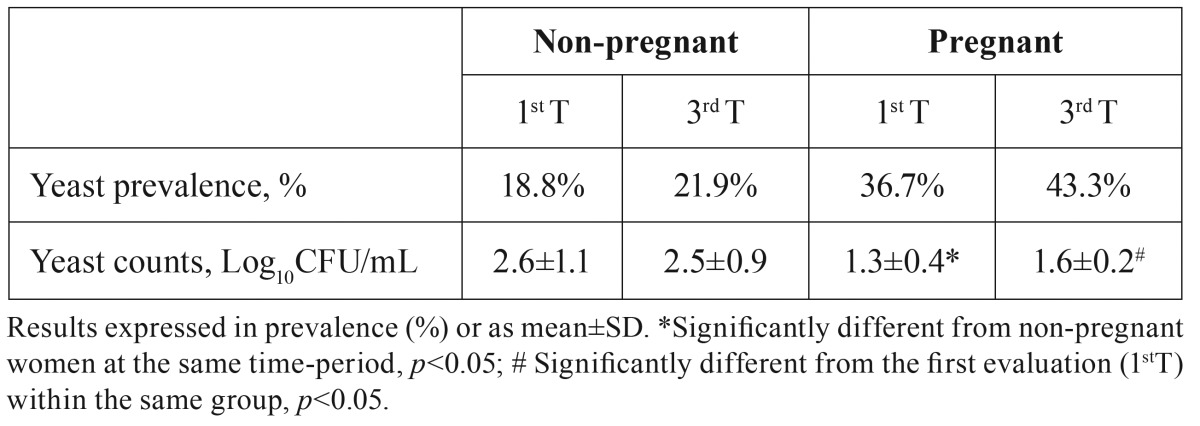
Oral yeast prevalence and counts in pregnant and in non-pregnant women.

**Figure 1:**
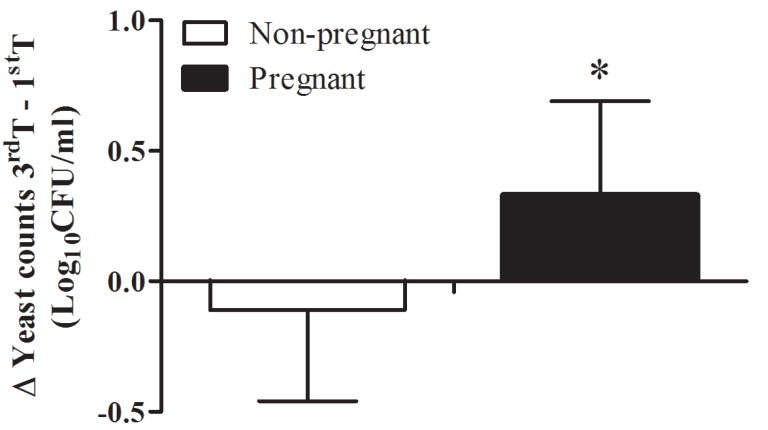
Average of variation in yeast load in colonized pregnant and non-pregnant women calculated by the difference between yeast counts (in Log10 CFU/ml) in 3rd and in 1st trimester. Bars represent mean and the error bars represent SD values. * Mann-Whitney U Test (*p* = 0.03).

When one looks to total yeast quantification (Log10CFU/mL) in individuals colonized with yeast, pregnant women presented lower yeast counts than non-pregnant women, attaining statistical significance in the 1st trimester (*p*=0.016). However, it was observed an increase in oral yeast counts from the 1st to the 3rd trimester in pregnant women (*p*=0.008). On the other hand, oral yeast counts did not differ from the 1st to the 3rd trimester in non-pregnant women (*p*=0.600). In figure 1 is depicted the differential of yeast counts between the first and second observation. This differential of yeast counts between the first and second observation attains statistical significance between pregnant and non-pregnant women.

Pregnant women colonized by yeast were all low or moderate yeast carriers whereas non-pregnant women include also asymptomatic high yeast carriers. The percentage of non-pregnant women with high yeast counts did not differ from the 1st to the 3rd trimester (12.5% vs. 9.4%, *p*>0.999). Within the group of high yeast carriers, there were no denture wearers, and no woman was on medication that could contribute to increase oral yeast counts.

## Discussion

Taking in consideration that maternal oral microbiome may represent the major contributor for the intrauterine microbiome (2), and that yeast may impact foetus development and pregnancy outcomes (12-15), we explore the changes of oral yeast colonization throughout pregnancy. Our data suggest that pregnant women may be more prone to oral yeast colonization than non-pregnant women.

In our study, the prevalence of pregnant women with oral yeast colonization was almost twice of that observed in non-pregnant women either in the first or in the third trimester; nevertheless this difference did not attain statistical significance. However, in the third trimester, this difference would be statistical significant if *p* < 0.10 was assumed. This result corroborates with a previous study from Fujiwara and colleagues (9) that shows higher *Candida* species prevalence in saliva from women in the middle and late pregnancy in comparison to the non-pregnant group. Although we did not identify yeast isolates, it is known that *Candida* sp. is the most frequent yeast colonizing the oral cavity (22). Furthermore, we observe an increase in oral yeast load from the first to the third trimester in pregnant women, but not in the control group, reinforcing the view that pregnancy may promote oral yeasts growth.

The promotion of yeast growth in the oral environment during pregnancy may be associated with the reduced oral pH observed in our pregnant women. In agreement with this suggestion are studies showing that *Candida*, the most prevalent yeast in oral cavity, is more adapted to acidic conditions (23).

A recent study showed a negative correlation between *Candida* load and the diversity of the salivary microbiome (24). In this view and taking in consideration the rise of yeast colonization during pregnancy, the oral microbiome may change during this period. In fact, some studies reported the proliferation of dominant bacteria species during pregnancy, namely *Porphyromonas gingivalis and Aggregatibacter actinomycetemcomitans* colonizing the gingival sulcus (9,16,25), and *Lactobacillus* sp. and *Streptococcus mutans* capable of metabolizing estradiol (16).

In scientific literature, the prevalence of oral yeast carriage is not consensual, ranging from 30% to 90% (22,26,27). The divergent results may be directly linked to the methods applied in different studies that can have distinctive sensitivities. It is important to note that the prevalence of oral yeast carriers in our control group is similar to other studies using standard microbiological culture techniques (9).

The oral yeast levels vary between individuals, and the scientific literature shows that a small percentage of individuals (~20%) present elevated yeast loads, without clinical signs of candidiasis (22). Although our results suggest that pregnancy promotes yeast colonization revealed by the higher prevalence of pregnant women colonized with oral yeast and an increase in oral yeast load throughout pregnancy, non-pregnant women presented higher yeast load than pregnant women. This apparent paradox may be due to the fact that high yeast carriers are exclusively present in non-pregnant group, and that can be explained by the sampling effect. Note that high yeast carriers presented yeast load > 2.8 Log10CFU/ml whereas all other individuals presented yeast load < 1.8 Log10CFU/ml.

In a study from 1980, higher values of oral *Candida* were suggested to be indicative of oral candidiasis, but an overall assessment of each individual should be performed (21). None high yeast carriers observed in our study presented clinical signs of candidiasis. Also, none of the evaluated factors known to contribute to elevated *Candida* colonization differ in the group of high yeast carriers, namely denture use, smoking habits and medication (28,29), so other non-evaluated factors must be involved.

In conclusion, our study provides evidence favouring the view that pregnancy may promote oral yeast growth and further suggests that this may be associated with an acidic oral environment.
